# Improving Wound Assessment Documentation Using a Structured Scoring Tool: A Closed-Loop Quality Improvement Project at Al Managil Teaching Hospital, Sudan

**DOI:** 10.7759/cureus.108110

**Published:** 2026-05-01

**Authors:** Rayan Hamza Mohamed Ahmedalgabri, Nada Abdelnaser Ahmed Alshorbagy, Amr Elbanna Mohammed Aly, Khaled Yousry Ibrahim Mansour, Saria Elsiddig Mohamed Ali, Alaa Elnour Yousif Mohamed, Israa Awad Ahmed Mohamed, Aisha Hassab Elrasoul Osman Mohammed, Mogahid Hamdan Adam Ahmed, Mustafa Mohamed, Aisha Alzain, Mohammed Abdelnour A Alfaki, Malaz Siddeg Hamed Younis, Rawan Tarig Mohamed Galaladein, Azza Ayoub Ali Elhadi, Heba Mahmoud Elgazar, Abdalmahmoud Asadig Kanan Ahmed, Asma Ahmed Osman Mohamed, Omar Abdelmajeed Maryoud Elbashir

**Affiliations:** 1 Dermatology, Regional Dermatology Training Centre, Moshi, TZA; 2 Surgery, Al Managil Teaching Hospital, Al Managil, SDN; 3 Emergency, Al-Shamal Specialized Hospital, Dongola, SDN; 4 General Surgery, Madina National Hospital, Medina, SAU; 5 General Surgery, University of Kordofan, El-Obeid, SDN; 6 Internal Medicine, Al Neelain University, Khartoum, SDN; 7 Emergency, Al Managil Teaching Hospital, Al Managil, SDN; 8 General Surgery, Sudan Medical Specialisation Board, Khartoum, SDN; 9 General Surgery, King Fahad Hospital, Al-Baha, SAU; 10 Surgery, King Saud Medical City, Riyadh, SAU; 11 Internal Medicine, Al Managil Teaching Hospital, Al Managil, SDN; 12 General Surgery, Al Managil Teaching Hospital, Al Managil, SDN

**Keywords:** documentation compliance, managil teaching hospital, quality improvement, wound assessment, wound-dress

## Abstract

Background

Incomplete wound assessment documentation can compromise clinical decision-making, continuity of care, and patient safety. Structured documentation tools have been proposed to standardize wound assessment practices and reduce variability. This quality improvement project evaluated the impact of implementing a structured wound assessment scoring tool on documentation completeness at Al Managil Teaching Hospital.

Methods

A closed-loop quality improvement project was conducted using two audit cycles. Fifty wound dressing records were reviewed during a two-week baseline assessment (Cycle 1). Following analysis of deficiencies, a structured wound assessment scoring tool (WOUND-DRESS) was introduced alongside focused staff education and reinforcement over a two-month implementation period. A second audit cycle was conducted over three months, reviewing 50 additional records. Compliance with predefined documentation standards was compared between cycles using chi-square testing.

Results

Significant improvements were observed across most documentation domains. Recording of wound exudate increased from 25/50 (50%) to 50/50 (100%) (p < 0.001), depth and tissue exposure from 14/50 (28%) to 50/50 (100%) (p < 0.001), and infection risk from 8/50 (16%) to 50/50 (100%) (p < 0.001). Calculation of total wound score improved from 3/50 (6%) to 50/50 (100%) (p < 0.001), and documentation of action taken increased from 21/50 (42%) to 50/50 (100%) (p < 0.001). Administrative variables also improved, although documentation of dressing type did not demonstrate significant improvement.

Conclusion

Implementation of a structured wound assessment scoring tool was associated with substantial improvements in documentation completeness, particularly in safety-critical assessment domains. Structured tools combined with targeted education and audit feedback can enhance standardization of wound documentation and support patient safety. Ongoing monitoring is required to sustain these improvements.

## Introduction

Wound assessment and documentation are fundamental components of clinical care that directly influence treatment decisions, monitoring of healing progression, and overall patient outcomes. High-quality documentation serves not only as a record of clinical findings and interventions but also as a communication tool that ensures continuity of care between healthcare professionals. Comprehensive wound documentation is recognized as a means to support clinical reasoning, justify treatment choices, and protect clinicians and healthcare facilities from medicolegal risks associated with incomplete records. Inadequate documentation has been shown to compromise both clinical decision-making and the legal defensibility of care provided [1,2].

Despite the acknowledged importance of detailed wound documentation, studies across hospital settings have consistently identified gaps in the completeness and quality of recorded wound assessments. In one audit evaluating nursing documentation within dressing charts, a large proportion of entries lacked key clinical details such as wound size, exudate type, and tissue characteristics, demonstrating significant deficiencies in routine practice [3]. Other literature highlights ongoing variation in assessment practices, with clinicians frequently focusing on technical dressing procedures without systematically documenting holistic wound characteristics, such as tissue viability and signs of infection [4].

To support standardization of care and reduce unwarranted variation, the use of structured wound assessment tools has been advocated. These tools aim to provide objective and reproducible criteria for evaluating wounds, facilitating more complete documentation and aiding clinical decision-making. However, evaluations of existing wound assessment instruments indicate that none fully meet all criteria required for optimal clinical utility, underscoring the challenge of selecting or adapting tools that are fit-for-purpose in diverse practice environments [5,6]. Moreover, evidence-based practice guidelines emphasize the value of systematic wound assessment as a cornerstone of effective wound management and consistent care delivery, although practical implementation in routine clinical settings remains limited [7].

Clinical audit and quality improvement methodologies are established approaches to measuring compliance with clinical standards, identifying areas for improvement, and evaluating the impact of targeted interventions. These methodologies provide a systematic framework that supports iterative cycles of evaluation and change, enabling healthcare teams to implement context-specific solutions and demonstrate measurable improvements in practice. Audit cycles that incorporate structured tools and feedback mechanisms have been shown to enhance adherence to documentation standards and improve clinical processes in various care domains [8].

At Al Managil Teaching Hospital, preliminary observations and informal reviews suggested inconsistency and incompleteness in wound assessment documentation, raising concerns about the potential for suboptimal dressing decisions and compromised patient safety. In response, this quality improvement project was designed to enhance the safety and standardization of wound assessment and dressing documentation. The general objective was to assess and improve compliance with established clinical documentation standards, and the specific objective was to determine whether implementation of a standardized wound assessment scoring system, accompanied by targeted education and audit feedback, would significantly improve the completeness and quality of wound documentation and associated dressing decisions over two audit cycles. The primary outcome was the proportion of wound dressing records demonstrating full compliance with predefined documentation standards.

## Materials and methods

Study design and framework

This study was conducted as a closed-loop quality improvement project using a two-cycle audit design structured around the Plan-Do-Study-Act (PDSA) framework [9]. The project aimed to evaluate baseline compliance with predefined wound assessment and documentation standards, implement a structured intervention to address identified gaps, and reassess performance to determine the effectiveness and sustainability of change.

Study setting

The project was carried out at Al Managil Teaching Hospital, a secondary-level teaching hospital providing medical and surgical inpatient services. Wound dressing procedures are routinely performed across inpatient wards by junior doctors and nursing staff as part of standard clinical care. At the time of the baseline assessment, documentation was paper-based, and no standardized wound assessment scoring system was in routine use.

Study population and sampling

The study population comprised adult inpatients who required wound dressing during the defined audit periods. Eligible cases included all documented wound dressing procedures performed in medical and surgical wards during each cycle. Pediatric patients and cases managed exclusively in outpatient settings were excluded. A consecutive sampling approach was adopted, whereby all eligible records during the specified timeframes were included to ensure representation of routine clinical practice and minimize selection bias.

Audit cycles and timeline

The quality improvement project was conducted over three phases. The first audit cycle was performed over a two-week period beginning in October, during which baseline compliance with wound assessment and documentation standards was evaluated without modification to existing practice. Following the analysis of baseline findings, a structured intervention was implemented over a two-month period. The intervention consisted of introducing a standardized wound assessment scoring system (WOUND-DRESS tool) based on a 0-2 scoring framework, delivering focused educational sessions for junior doctors and relevant ward staff, clarifying safety-critical documentation elements and escalation criteria, and reinforcing adherence through visual guidance materials and supervisory feedback during ward rounds.

The WOUND-DRESS scoring system was developed locally by the project team as a structured documentation framework to standardize wound assessment recording. The tool incorporates key wound assessment domains derived from widely accepted wound assessment principles described in published wound care literature and clinical practice recommendations [5-7]. As a locally developed quality improvement documentation tool, the WOUND-DRESS framework is not a proprietary instrument and does not require licensing for clinical or academic use.

The educational intervention included structured teaching sessions delivered to junior doctors and ward staff over the two-month implementation period. These sessions included orientation to the WOUND-DRESS tool, a detailed explanation of scoring criteria, and case-based examples demonstrating its application in clinical practice. Reinforcement was provided through visual aids displayed in clinical areas and ongoing feedback during routine ward rounds.

The second audit cycle was conducted over a three-month period following completion of the intervention phase. The same inclusion criteria, data collection methodology, and evaluation standards were applied in both cycles to ensure comparability.

Standards and compliance criteria

Predefined standards were developed in accordance with infection control principles, structured wound assessment recommendations, and hospital safety requirements. Each wound dressing entry was expected to include patient identifiers, date and time of assessment, and a structured wound evaluation using the standardized 0-2 scoring system assessing exudate, depth or tissue exposure, infection risk, mechanical or environmental risk, pain or sensitivity, and patient self-care capacity.

The standardized 0-2 scoring system was defined for each domain to reflect increasing severity. A score of 0 indicated normal or low-risk findings (e.g., no exudate, superficial wound without tissue exposure, no signs of infection, minimal mechanical risk, no pain, and full self-care capacity). A score of 1 indicated moderate findings (e.g., mild to moderate exudate, partial tissue involvement, possible or early signs of infection, moderate mechanical or environmental risk, tolerable pain, or partial dependence in self-care). A score of 2 indicated severe or high-risk findings (e.g., heavy exudate, deep wound with significant tissue exposure, clear signs of infection, high mechanical or environmental risk, severe pain, or inability to perform self-care).

A total wound score was required to be calculated in each case. Dressing selection was expected to correspond to the calculated total score, and mandatory escalation was required if infection risk or tissue depth was scored at the highest severity level. Documentation of the assessing professional was also required. Safety-critical elements - specifically total score calculation, escalation documentation, and action taken - were predefined to achieve 100% compliance. Compliance was defined as complete and accurate documentation of each required element within the patient’s medical record.

Data collection

Data were collected through a structured retrospective review of patient medical records and wound dressing documentation charts during each audit cycle. A standardized data extraction tool was developed prior to the commencement of the first cycle to ensure consistency in data collection. Reviewers were trained to apply uniform interpretation criteria when assessing documentation completeness. Two independent reviewers were trained to apply uniform interpretation criteria when assessing documentation completeness. Reviewers applied predefined criteria using the standardized data extraction tool to ensure consistency across both audit cycles. Formal inter-rater reliability testing was not conducted, which is acknowledged as a limitation of the study.

Each record was assessed for the presence or absence of required elements. All data were anonymized at the point of collection, and serial identifiers were assigned to maintain confidentiality.

Outcome measures

The primary outcome measure was the proportion of wound dressing records demonstrating full compliance with the predefined documentation standards. Secondary outcomes included compliance rates for individual wound assessment components, the proportion of cases in which the total wound score was accurately calculated, the frequency of documented escalation when indicated, and the extent to which dressing selection was aligned with the calculated total score.

Statistical analysis

Data were entered into a secure database and analyzed using descriptive statistical methods. Categorical variables were summarized as frequencies and percentages. Comparisons between baseline and post-intervention compliance rates were performed using chi-square testing to evaluate the statistical significance of observed differences. A p-value of less than 0.05 was considered statistically significant.

Ethical and governance considerations

This project was conducted as a service evaluation and quality improvement initiative aimed at enhancing documentation practices and patient safety. No changes were made to clinical treatment beyond standard care. All data were anonymized prior to analysis, and patient confidentiality was maintained throughout. Institutional approval was obtained from the Al Managil Teaching Hospital (approval no: WD-05-1777) before commencement of the project.

## Results

A total of 100 wound dressing records were reviewed, with 50 records included in each audit cycle.

Patient identification and administrative variables

Documentation of patient identifiers improved significantly following the intervention. Recording of the patient’s name increased from 39/50 (78.0%) in Cycle 1 to 50/50 (100%) in Cycle 2 (χ² = 10.215, p = 0.0014). Documentation of age improved from 38/50 (76.0%) to 50/50 (100%) (χ² = 11.458, p = 0.0007), while diagnosis increased from 36/50 (72.0%) to 50/50 (100%) (χ² = 14.037, p = 0.0002).

Hospital number documentation demonstrated a marked improvement from 7/50 (14.0%) at baseline to 33/50 (66.0%) post-intervention (χ² = 26.042, p < 0.001). Documentation of bed number also improved significantly from 4/50 (8.0%) to 17/50 (34.0%) (χ² = 8.680, p = 0.0032). Recording of date and time increased from 31/50 (62.0%) to 50/50 (100%) (χ² = 21.053, p < 0.001). However, documentation of ward location did not show a statistically significant improvement, increasing modestly from 29/50 (58.0%) to 33/50 (66.0%) (χ² = 0.382, p = 0.5365).

Wound assessment components

Substantial improvements were observed across all wound assessment domains introduced through the structured scoring tool. Documentation of wound exudate increased from 25/50 (50.0%) to 50/50 (100%) (χ² = 30.720, p < 0.001). Recording of depth and tissue exposure improved from 14/50 (28.0%) to 50/50 (100%) (χ² = 53.168, p < 0.001), while documentation of infection risk rose from 8/50 (16.0%) to 50/50 (100%) (χ² = 69.007, p < 0.001).

Mechanical and environmental risk documentation demonstrated one of the largest absolute improvements, increasing from 3/50 (6.0%) at baseline to 50/50 (100%) post-intervention (χ² = 84.946, p < 0.001). Similarly, documentation of patient self-care capacity improved from 7/50 (14.0%) to 50/50 (100%) (χ² = 71.971, p < 0.001), and recording of pain and sensitivity increased from 22/50 (44.0%) to 50/50 (100%) (χ² = 36.161, p < 0.001).

Safety-critical documentation

Calculation of the total wound score, a predefined safety-critical element, improved from 3/50 (6.0%) in Cycle 1 to 50/50 (100%) in Cycle 2 (χ² = 84.946, p < 0.001). Documentation of action taken in response to wound assessment findings increased from 21/50 (42.0%) to 50/50 (100%) (χ² = 38.077, p < 0.001). These findings demonstrate complete compliance with core safety elements following implementation of the WOUND-DRESS tool.

Additional documentation variables

Documentation of the assessing staff improved from 19/50 (38.0%) to 30/50 (60.0%), reaching statistical significance (χ² = 4.002, p = 0.0455). In contrast, documentation of dressing type and medications decreased from 24/50 (48.0%) to 17/50 (34.0%), and this difference was not statistically significant (χ² = 1.488, p = 0.2225) (Table 1).

**Table 1:** Completeness of wound assessment and wound dressing documentation before and after implementation of the WOUND-DRESS scoring tool at Al Managil Teaching Hospital (Cycle 1 vs. Cycle 2; n = 50 per cycle). Values are presented as n (%) for records in which the item was documented (“mentioned”). Cycle 1 represents the baseline assessment performed over two weeks in October, while Cycle 2 represents the post-intervention re-audit conducted over three months following a two-month implementation period. Comparisons between cycles were performed using the chi-square test for independence on 2×2 contingency tables (documented vs. not documented). A two-sided p-value < 0.05 was considered statistically significant. The WOUND-DRESS structured assessment tool is a locally developed documentation framework based on established wound assessment principles described in published wound care literature and clinical practice recommendations [5-7].

Variable	Cycle 1, n (%)	Cycle 2, n (%)	χ²	p-value
Name	39 (78.0)	50 (100.0)	10.215	0.0014
Age	38 (76.0)	50 (100.0)	11.458	0.0007
Diagnosis	36 (72.0)	50 (100.0)	14.037	0.0002
Hospital number	7 (14.0)	33 (66.0)	26.042	<0.001
Ward	29 (58.0)	33 (66.0)	0.382	0.5365
Bed	4 (8.0)	17 (34.0)	8.680	0.0032
Date and time	31 (62.0)	50 (100.0)	21.053	<0.001
Wound exudate	25 (50.0)	50 (100.0)	30.720	<0.001
Depth and tissue exposure	14 (28.0)	50 (100.0)	53.168	<0.001
Infection risk	8 (16.0)	50 (100.0)	69.007	<0.001
Mechanical/environmental risk	3 (6.0)	50 (100.0)	84.946	<0.001
Pain and sensitivity	22 (44.0)	50 (100.0)	36.161	<0.001
Patient self-care capacity	7 (14.0)	50 (100.0)	71.971	<0.001
Total score	3 (6.0)	50 (100.0)	84.946	<0.001
Action taken	21 (42.0)	50 (100.0)	38.077	<0.001
Assessing staff	19 (38.0)	30 (60.0)	4.002	0.0455
Dressing type and medications	24 (48.0)	17 (34.0)	1.488	0.2225

## Discussion

This closed-loop quality improvement audit found that implementing a structured wound assessment and documentation tool was associated with statistically significant improvements in the completeness of wound care records at Al Managil Teaching Hospital. Baseline data showed considerable gaps in critical documentation elements, a problem that has been well documented in the literature, where audits often report incomplete assessments, absent clinical characteristics, and inconsistent recording of wound findings [10]. Structured approaches to wound assessment can provide clinicians with clear, objective criteria for documenting key wound characteristics that are essential for informed clinical decision-making, continuity of care, and outcome monitoring [5].

Systematic wound assessment encompasses more than recording surface appearance; it requires detailed evaluation of wound depth, exudate volume and type, tissue viability, and infection risk. These components have repeatedly been identified as being crucial for tailoring treatment plans and predicting healing trajectories [11]. By integrating such criteria into a structured documentation tool, clinicians in this audit were more likely to capture necessary details consistently, reducing omissions that compromise patient care. Improvements across all assessed wound domains in Cycle 2 reflect the impact of the structured tool, aligning with global calls for standardized documentation methodologies in wound practice [6].

The marked increase in safety-critical documentation elements, including total wound score calculation and documentation of action taken, is notable. Complete and accurate documentation facilitates not only clinical reasoning but also legal defensibility and quality assurance processes. Comprehensive records allow multidisciplinary teams to interpret wound progression and adapt plans accordingly, enhancing patient safety and care continuity [12]. Furthermore, clinical audit itself is a recognized quality improvement strategy, enabling teams to measure adherence to standards, identify gaps, implement change, and reassess performance in a cyclical manner [13]. Effective wound care also depends on clear communication and documentation within multidisciplinary teams to ensure coordinated management and continuity of care [14]. In addition, structured quality improvement approaches such as the PDSA cycle provide a systematic framework for evaluating healthcare practices and implementing iterative improvements in clinical processes [15].

Despite these improvements, several documentation domains did not demonstrate equivalent progress. Specifically, documenting dressing type and medications did not improve, suggesting that the intervention was more effective at improving assessment than treatment documentation. This mirrors findings in other settings where structured assessment tools did not automatically translate to better recording of downstream clinical actions without linked prompts or templates for treatment documentation [16]. Moreover, recording of the assessing staff - an element related to accountability - improved only modestly, highlighting the need for targeted emphasis on professional identification in future interventions.

This study has several strengths. It employed a closed-loop audit design using the PDSA framework, enabling systematic evaluation of baseline practice, implementation of targeted interventions, and reassessment of outcomes. The use of a structured, context-specific documentation tool combined with education and audit feedback resulted in consistent and measurable improvements across multiple clinically relevant domains. Additionally, the inclusion of clearly defined standards and objective outcome measures enhances the reproducibility of the project in similar healthcare settings.

However, several limitations should be considered. This was a single-center quality improvement project, which may limit generalizability to other institutions with different clinical workflows. The study primarily evaluated documentation practices rather than direct clinical outcomes such as wound healing rates or infection reduction. Furthermore, data collection relied on retrospective record review, which may be subject to observer bias and does not fully capture actual clinical practice beyond what was documented. The relatively short post-intervention follow-up period also limits the assessment of the long-term sustainability of the observed improvements.

The sustainability of these improvements beyond the immediate post-intervention period remains unclear. Effective documentation practices require ongoing reinforcement through continuous training, regular audit cycles, and embedding structured tools within routine workflows. Studies suggest that without sustained effort, initial gains in documentation quality may regress over time [8]. Future research should include longitudinal follow-ups and explore the integration of electronic tools, prompts, and feedback systems to support clinicians in maintaining high documentation standards.

In conclusion, the implementation of a structured wound assessment and documentation tool significantly enhanced the completeness of wound care documentation in this setting. These improvements support more reliable clinical decision-making and strengthen patient safety through better communication and comprehensive records. Continued refinement of assessment and treatment documentation processes, coupled with sustained quality improvement practices, will be essential to maintain and extend these gains.

## Conclusions

Implementation of a structured wound assessment and documentation scoring tool was associated with statistically significant improvements in the completeness of wound assessment documentation. Core wound assessment domains and safety-critical elements, including total score calculation and documentation of action taken, demonstrated marked improvement following the intervention.

These findings support the use of structured documentation frameworks, combined with targeted education and audit feedback, to enhance standardization of wound assessment and strengthen patient safety practices. Continued monitoring and reinforcement are required to sustain and consolidate these gains.
